# Chemotherapy with 5-fluorouracil, cisplatin and streptozocin for neuroendocrine tumours

**DOI:** 10.1038/sj.bjc.6605618

**Published:** 2010-03-16

**Authors:** N C Turner, S J Strauss, D Sarker, R Gillmore, A Kirkwood, A Hackshaw, A Papadopoulou, J Bell, I Kayani, C Toumpanakis, F Grillo, A Mayer, D Hochhauser, R H Begent, M E Caplin, T Meyer

**Affiliations:** 1 Department of Oncology, UCL Medical School, Royal Free Campus, London, UK; 2 Cancer Research UK and UCL Cancer Trials Centre, London, UK; 3 Department of Radiology, Royal Free Hospital, London, UK; 4 Department of Radiology, University College Hospital, London, UK; 5 Department of Gastroenterology, Royal Free Hospital, London, UK; 6 Department of Pathology, UCL Medical School, Royal Free Campus, London, UK; 7 Department of Oncology, UCL Cancer Institute, London, UK

**Keywords:** chemotherapy, NETs, Ki-67, mitotic index

## Abstract

**Background:**

The role of chemotherapy for neuroendocrine tumours remains controversial and there is no standard regimen.

**Method:**

We report the outcome for a consecutive series of chemonaive patients with metastatic or locally advanced neuroendocrine tumours treated with a combination of 5-fluorouracil (500 mg m^−2^), cisplatin (70 mg m^−2^) and streptozocin (1000 mg m^−2^) (FCiSt) administered three weekly for up to six cycles. Patients were assessed for radiological response, toxicity and survival.

**Results:**

In the 79 patients assessable for response, treatment with FCiSt was associated with an overall response rate of 33% (38% for pancreatic primary sites and 25% for non-pancreatic primary sites). Stable disease occurred in a further 51%, with progression in 16%. The median time to progression was 9.1 months and median overall survival was 31.5 months. The most common grade 3–4 toxicity was neutropaenia (28% patients) but grade 3–4 infection was rare (7%). The most frequent non-haematological grade 3–4 toxicity was nausea and vomiting (17%). Prognostic factors included Ki-67, mitotic index, grade and chromogranin A, whereas response to chemotherapy was predicted by mitotic index, grade and *α*-fetoprotein.

**Conclusions:**

FCiSt is an effective regimen for neuroendocrine tumours with an acceptable toxicity profile. Grade and mitotic index are the best predictors of response.

Neuroendocrine tumours (NETs) are a heterogeneous group of malignancies that arise from various sites in the body, most commonly the gastrointestinal (GI) tract. The clinical course varies from a highly aggressive disease with a median survival of around 6 months in patients with metastatic high-grade tumours, to a more indolent process in which patients live with their disease for up to 20 years (Pape *et al*, 2008a, 2008b; Yao *et al*, 2008). Recent data suggest that the incidence of NETs has increased fivefold over the past 30 years and is now 5.25 per 100 000. Moreover the long survival times mean that the prevalence of NETs is now greater than that of other GI tumours including pancreatic and oesophagogastric cancers (Yao *et al*, 2008).

Surgery is the only curative intervention, but in the majority of cases metastasis has already occurred at the time of presentation and palliative measures are required. For functional tumours, hormone-related symptoms can be controlled with somatostatin analogues, although whether somatostatin analogues influence tumour growth is not clear. Loco-regional interventions including embolisation, ablation or debulking surgery have also been used to ameliorate symptoms associated with hormone production.

A variety of systemic chemotherapy regimens have been explored (Toumpanakis *et al*, 2007) but there is no accepted standard of care. The initial studies by Moertel *et al* (1980, 1992) using streptozocin in combination with fluorouracil or doxorubicin in pancreatic NETs gave encouraging response rates of over 60% based on reduction of hepatomegaly and biochemical response. However subsequent studies applying more rigorous objective response criteria report response rates between 6 and 45% for pancreatic NETs (Eriksson *et al*, 1990; Cheng and Saltz, 1999; Kouvaraki *et al*, 2004) and 15–30% for non-pancreatic NETs (Engstrom *et al*, 1984; Bukowski *et al*, 1987; Sun *et al*, 2005). The combination of cisplatin and etoposide has also been evaluated, and high response rates have been reported for poorly differentiated tumours (Moertel *et al*, 1991; Mitry *et al*, 1999; Fjallskog *et al*, 2001). These studies have also included a small number of patients with well-differentiated endocrine cancers (WDECs) who had failed previous chemotherapy and achieved response rates ranging from 9 to 45%. However cisplatin has never been systematically tested as first-line therapy in a large series of WDECs.

Here we report for the first time, the clinical outcome of a series of 82 consecutive patients with advanced NETs prospectively collected over a period of 8 years, who were all treated with the novel combination: 5-fluorouracil (500 mg m^−2^), cisplatin (70 mg m^−2^) and streptozocin (1000 mg m^−2^) (FCiSt) on day 1 of a three weekly cycle. Patients were assessed for radiological response, toxicity and survival, and prognostic and predictive factors associated with response to chemotherapy were elucidated.

## Patients and methods

### Patients

Patients with advanced NETs treated with FCiSt chemotherapy at the Royal Free Hospital were identified from a prospective database. Between May 1999 and April 2008, 98 consecutive patients were treated of which 82 were included in the analysis. Reasons for exclusion were chemotherapy given with adjuvant intent (*n*=2), previous chemotherapy or chemoembolisation (*n*=12), no evaluable imaging or clinical follow-up (*n*=2). Patients who had undergone external beam radiotherapy earlier were included in the study, provided that disease was measured outside the radiation field. Patients with tumours of all primary sites and with unknown primary tumours were included in the analysis. Two patients received subcutaneous octreotide therapy concurrently with chemotherapy. Indications to commence chemotherapy were radiological progression (*n*=17), tumour grade (*n*=25), progressive symptoms related to increasing tumour burden (rather than hormone secretion) (*n*=36) or intention of downstaging the disease for surgery (*n*=4).

### Chemotherapy

FCiSt chemotherapy consisted in order: calcium folinate 45 mg over 2 h, 5-fluorouracil 500 mg m^−2^ by slow bolus, streptozocin 1000 mg m^−2^ in 1000 ml 0.9% saline (Nsaline) over 2 h, mannitol 20 g in 200 ml Nsaline over 30 min, cisplatin 70 mg m^−2^ in 1000 ml Nsaline over 2 h followed by post-hydration. Chemotherapy was administered every 21 days for a maximum of 6 cycles. For patients with glomerular filtration rate (GFR), assessed by ^51^Cr-EDTA clearance, less than 60 ml min^−1^, either at baseline or that developed during chemotherapy, cisplatin was substituted with carboplatin AUC 5 mg ml^−1^ min^−1^ (Calvert formula) referred to as FCaSt. Cycles of chemotherapy were delayed until recovery of neutrophil count ⩾1.0 × 10^9^ per litre and platelet count ⩾100 × 10^9^ per litre. Doses of all drugs were reduced by 20% following grade 3 non-haematological toxicity or treatment delay due to myelosuppression. Patients who had not progressed after three cycles of chemotherapy continued to six cycles, in the absence of unacceptable toxicity.

### Response and toxicity assessment

Baseline scans were performed within 6 weeks before starting chemotherapy. Response was assessed by CT scan after three cycles of chemotherapy, with patients continuing to six cycles of chemotherapy in the absence of progression. Further response assessment was by CT scan after completion of chemotherapy, and at three monthly intervals until progression. Three patients were not assessable for response after receiving two, three and six cycles of chemotherapy respectively. Response was determined by two independent radiologists using RECIST criteria, with best response reported without a subsequent confirmation scan. Time to response (TTR) was recorded as the time from the date of commencing chemotherapy to demonstration of objective response assessed by independent review, and duration of response from the date of scan confirming objective response to disease progression. Progression-free survival (PFS) was recorded as the time from the date of commencing chemotherapy to demonstration of objective disease progression or death. Overall survival (OS) was recorded as the time from the date of commencing chemotherapy to the patient's death. Adverse effects were recorded prospectively with each cycle of chemotherapy, and reported using CTCAE version 3.0 (http://ctep.cancer.gov).

### Pathology and tumour assessment

Patients were diagnosed as having an NET both morphologically and immunohistochemically. Ki-67 index was assessed by an independent pathologist using the MIB1 antibody and counting the percentage of positive tumour cells in 2000 neoplastic cells in the area of highest labelling, and mitotic index (MI) was reported as the number of mitoses per 10 high-power fields (HPFs) in areas of highest mitotic activity. Tumours were graded using the proposed grading system of Rindi *et al* (2006). Briefly tumours were graded as grade 1 (low) with MI <2 per 10 HPFs and/or Ki-67 ⩽2%, grade 2 (intermediate) with MI 2–20 per 10 HPFs and/or Ki-67 3–20% and grade 3 (high) with MI >20 per 10 HPFs and Ki-67 >20%. Tumour somatostatin receptor expression was determined by indium octreotide scintigraphy, urinary 5-hydroxyindoleacetic acid (5HIAA) was assessed by 24 h urine collection using standard laboratory methods, and chromogranin A (CGA) was measured using standard laboratory methods and reported as abnormal at >60 U l^−1^. Baseline blood markers were measured within 3 months of the start of chemotherapy.

### Statistical analysis

Statistical analyses were performed using STATA version 10 (Stata Corp. LP, College Station, TX, USA). Median TTR, duration of response, time to progression and OS were calculated using Kaplan–Meier survival analysis.

We explored potential markers that were predictive of response in relation to treatment, or prognostic in terms of overall or PFS. Ki-67 index and MI were initially examined as continuous data but because they were not normally distributed (even when transformed onto a logarithmic scale) they were divided up into approximate tertiles and analysed as categorical variables. CGA was analysed in two groups; above and below the upper limit of the normal (ULN) range. Tumour markers were analysed in two groups; above and below 1.5 × ULN, which we have previously reported as a prognostic cut off for *α*-fetoprotein (AFP) and human chorionic gonadotropin (HCG; Shah *et al*, 2008). Fisher's exact test and *χ*^2^-test for trend were used for predictive markers and data were reported for all eligible patients. Cox regression analyses allowing for grade, performance status, primary site (pancreatic or non-pancreatic) and age at the start of chemotherapy were performed for each factor and a hazard ratio and 99% confidence interval were calculated. 99% was chosen to allow for examination of several factors.

## Results

### Patient characteristics

We treated 82 patients with a median age of 55 years (range 22–81 years) (Table 1). Primary tumour site was pancreatic in 49 patients (60%), GI tract in 9 patients (11%), lung in 8 patients (10%), ovarian in 1 patient and unknown in 15 patients (18%). Metastatic disease was present in 89% of patients and the liver was the most common secondary site occurring in 76%. Previous systemic treatment included subcutaneous octreotide therapy, ^131^I MIBG (*n*=4), ^90^Y DOTA octreotide radionucleotide therapy (*n*=3), arterial embolisation, interferon-*α*, external beam radiotherapy and 57 patients (70%) had no prior therapy (Table 1). At least three cycles of chemotherapy were given in 93% cases and 72% received at least six cycles. Six patients were treated with carboplatin AUC 5 due to a low GFR at baseline (*n*=5) and poor cardiac function precluding appropriate pre-hydration (*n*=1). A further six patients were switched from cisplatin to carboplatin because of emergent cisplatin-related toxicity.

### Adverse events

Chemotherapy, in general, was well tolerated with adverse effects detailed in Table 2. The most common toxicity was fatigue occurring in 73% of patients but with grade 3 fatigue in only 7% patients. Grade 3 or 4 side effects were uncommon, with the highest incidence being neutropaenia (28%) although grade 3/4 infection only occurred in 7%. There was no grade 3/4 renal toxicity and only 2% had grade 3/4 neuropathy. In contrast to doxorubicin-containing regimens FCiSt did not cause hair loss. There were no toxic deaths, although one patient died on treatment due to disease progression.

### Response assessment

Of the 82 patients, 79 were assessable for response. The response rate for tumours of pancreatic origin was 38% (18 out of 47 patients) whereas that for non-pancreatic tumours was 25% (8 out of 32 patients) (Table 3). One patient with a grade 3 bronchial NET achieved a partial response after six cycles and went on to have his primary tumour and involved mediastinal lymph nodes resected. On histological examination there was no viable remaining tumour and he remains well and disease free 5 years after resection. Overall, objective response rate was 33% (*n*=26), with stable disease as best response in 51% (*n*=40), and progressive disease in 16% (*n*=13). Stable disease lasting more than 6 months occurred in 39% (*n*=31) of patients. The median TTR was 20 weeks (range 7–47 weeks) and the median duration of response was 36 weeks. Delayed response was observed in five patients (19% of responding patients), with RECIST criteria for response not being met at the end of treatment CT scan but reached on subsequent follow-up CT scans while off treatment.

### Prediction of response

We examined factors that predicted for response to chemotherapy (Table 3). We found no statistical difference in response rates between tumours with pancreatic primary and non-pancreatic primary sites (38 *vs* 25% respectively, *P*=0.24, Fisher's exact test). There was some evidence to suggest that histological grade predicted response to chemotherapy, with a higher response rate with higher histological grade (*P*=0.02, *χ*^2^-test for trend). Of the five patients with delayed response to chemotherapy one had a high-grade tumour, three had intermediate-grade tumour and one had low-grade tumour.

We also assessed how well proliferative fraction, assessed with Ki-67, or MI predicted response. Overall there was a moderately strong correlation between MI and Ki-67 (*r*=0.68, *P*<0.0001, Spearman's correlation coefficient). We found the MI to be significantly associated (*P*=0.008, *χ*^2^-test for trend) with response to chemotherapy, with response rates increasing from 15% for MI 0–1 to 55% for MI ⩾5.

There was some evidence to suggest that Ki-67 index may also predict response to chemotherapy (Table 3), although the difference in response rate between low, medium and high tertiles did not quite reach statistical significance (*P*=0.019, *χ*^2^-test for trend). Similarly there was some evidence that poorly differentiated tumours had a higher response rate than well-differentiated tumours (*P*=0.046).

Of the tumour markers examined, there was some evidence that AFP above 16 ng ml^−1^ may be predictive of response (64 *vs* 25% *P*=0.02) whereas elevated *β-*HCG, CA19-9 and CGA were uninformative.

### Survival

**Figure 1:**
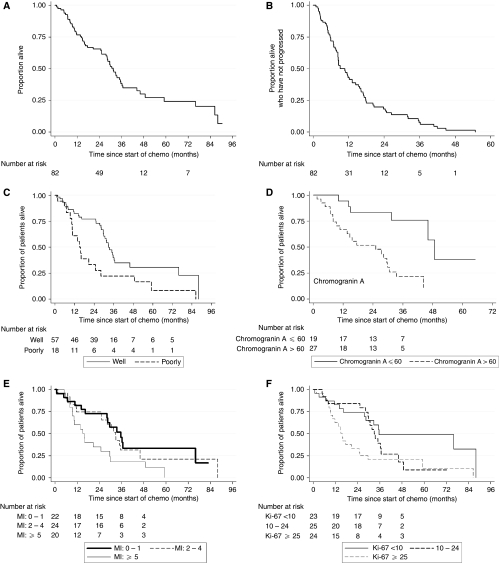
Overall survival (**A**) and progression-free survival (**B**). Overall survival according to differentiation status (**C**), normal or elevated serum chromogranin A (**D**) mitotic index (MI) (**E**) Ki-67 index (**F**).

Patients were followed up for a median of 45 months. The 1- and 2-year survival was 76.7% (95% CI 66–84.5) and 65.5% (95% CI 54.1–74.7) respectively and the median OS was 31.5 months (Figure 1A). The 1- and 2-year PFS was 43.0% (95% CI 31.8–53.6) and 18.3% (95% CI 10.3–28.1) and median PFS was 9.1 months (Figure 1B). There was some evidence to suggest that performance status (>1), non-pancreatic primary and poorly differentiated tumours (Figure 1C) may have been associated with a worse OS whereas age was not prognostic (Table 4). Hazard ratios and 99% CIs, adjusted for age, performance status, primary site and grade, were calculated for each potential prognostic factor (Table 5). A baseline CGA of >60 U l^−1^ appeared to be strongly predictive of a worse OS (Figure 1D), with the hazard ratio for patients in the higher group reaching more than six times that of the patients in the lower group (Table 5). Ki-67 and MI were adjusted for performance status, age and primary site only (as tumour grade is based on their values). There was also evidence to suggest that both a high MI (⩾5) and Ki-67 (>25%) were strongly associated with poorer OS although neither quite reached statistical significance (*P*=0.017, for both; Table 5; Figures 1D and E). Elevated *α*-fetoprotein, *β-*HCG and CA19-9 levels do not seem to be prognostic.

## Discussion

In this study we report encouraging anti-tumour activity with acceptable toxicity using a novel combination of 5-fluorouracil, streptozocin and cisplatin for the treatment of advanced NETs. The overall disease control rate at 6 months was 72% with a median time to progression of 9.1 months and a median OS of 31.5 months, all of which compares favourably with previously reported large streptozocin-based regimens (Moertel *et al*, 1980, 1992; Engstrom *et al*, 1984; Kouvaraki *et al*, 2004; Sun *et al*, 2005). Pancreatic NETS had a higher response rate than non-pancreatic NETs (38 *vs* 25%) and although this was not statistically significant, the trend is consistent with other studies. Importantly we found that one-fifth of responding patients had a delayed response, achieving stable disease at their end of treatment scan but partial response during subsequent follow-up while off treatment. A similar observation was made by Kouvaraki *et al* (2004) who reported a comparable median TTR of 4 months for fluorouracil, doxorubicin and streptozocin in the treatment of pancreatic NETs. However in that series chemotherapy was continued for up to 15.5 months and the authors argued that late response was a justification to continue chemotherapy until progression. In contrast our study data suggest that delayed response occurs even in the absence of ongoing chemotherapy and reflects the distinct biology of these tumours. This finding underlines the importance of longer-term follow-up imaging to define response accurately.

The FCiSt regimen was generally well tolerated. In contrast to previous studies, we have used an outpatient, 1 day, three weekly schedule using 1000 mg m^−2^ streptozocin rather than the 4–500 mg m^−2^ 5-day regimen, thereby improving patient convenience without compromising efficacy. It is notable that the combination of two nephrotoxic agents, streptozocin and cisplatin, used according to our schedule did not cause any grade 3–4 renal toxicity. Concern about renal toxicity for cisplatin in the field of NETs was raised by two studies (Moertel *et al*, 1991; Fjallskog *et al*, 2001) in which cisplatin was administered as a continuous infusion 45 mg m^−2^ per day for 2 days. Some patients in these studies had already received high-dose streptozocin and had documented renal impairment. By contrast our patients were chemonaive and were required to have a baseline GFR above 60 ml min^−1^ as outlined in the methods. Similar to the study by Mitry *et al* (1999), who reported minimal renal toxicity for a 2 h infusion of cisplatin at a dose of 100 mg m^−2^, we experience minimal toxicity using a short infusion at 70 mg m^−2^.

The prognostic factors associated with survival in patients with NETs have been well defined and include grade, stage, primary site, sex, age and race (Yao *et al*, 2008) and, in patients treated with chemotherapy, the extent of liver metastasis (Kouvaraki *et al*, 2004). In our selected cohort of patients CGA was found to be prognostic for OS and there was evidence to suggest that Ki-67 index and MI may also be prognostic but in this sample of patients, they did not quite reach statistical significance. Unusually, patients with a pancreatic primary tumour had a better survival than those with a non-pancreatic primary tumour. The likely explanation for this observation is that in our series, selected for chemotherapy, 30% of patients with non-pancreatic primary tumour had high-grade tumours whereas for patients with pancreatic primary tumour the proportion was only 10% and grade was a dominant prognostic factor (Pape *et al*, 2008b; Yao *et al*, 2008).

Less well defined in the literature are the factors that predict response to chemotherapy. Assessment of the proliferation fraction as measured by MIB1 staining of Ki-67 >10% has been proposed as a means of selecting patients for cytotoxic treatment but it is acknowledged that clear evidence for this strategy is lacking (Vilar *et al*, 2007). We have used our large, homogeneously treated, consecutive series to examine for predictors of response. In our analysis there was a trend for response rate to increase with a higher Ki-67 index but even in those with Ki-67⩽9% the response rate was 18%. Hence if Ki-67 is to be used to select patients for chemotherapy the cut off remains to be defined but our study data suggest that it should be less than 10%. MI and tumour grade were good predictors of response with higher-grade tumours and those with a high mitotic rate having a higher response rate. To the best of our knowledge this is the first systematic analysis of MI and Ki-67 index as predictors of response and prognosis for NETs in patients treated with chemotherapy.

We have previously reported that elevations of AFP and *β*-HCG (>1.5 × ULN) are associated with a poorer survival in NETs (Shah *et al*, 2008). In contrast neither of these markers was prognostic in the current cohort treated with chemotherapy. Intriguingly an elevated AFP may be associated with a higher response rate and one can speculate that treating these patients with chemotherapy improves their survival such that the prognostic value of the baseline measurement is obscured. Neither serum CGA, nor urinary 5HIAA nor somatostatin receptor expression had any predictive value.

Importantly, we were unable to identify a group of patients who did not respond to chemotherapy. In this series, 14% (1 out of 7) of the few patients with low-grade tumours responded to chemotherapy, and 15% (3 out of 20) of tumours with an MI of <2 (low-grade MI) responded, challenging the view that these tumours are resistant to chemotherapy. However, there are a number of factors that should be considered in interpreting this data. Potentially this finding may reflect tumour heterogeneity not captured by percutaneous biopsy, with the response in ‘low-grade’ tumours reflecting unsampled higher-grade tumour that responds to chemotherapy. It is also possible that histologically low-grade tumours at diagnosis have become higher-grade tumours by the time treatment is initiated. Finally, the late responding nature of many low/intermediate-grade tumours, which we have identified in this study, suggests that chemosensitivity of these tumours may have previously been underestimated.

Non-streptozocin-based chemotherapy regimens have been explored in NETs and while agents such as paclitaxel (Ansell *et al*, 2001) and gemcitabine (Kulke *et al*, 2004) appear to be ineffective, there are encouraging data for temozolamide in uncontrolled phase II trials (Kulke *et al*, 2006; Ekeblad *et al*, 2007). A significant difficulty in comparing reports for different chemotherapy regimens in NETs relates to heterogeneity of this patient population. Survival times can be as short as 5 months for poorly differentiated metastatic tumours or as long as 223 months for those with localised well-differentiated tumours (Yao *et al*, 2008). Here we have shown the predictive value of grade, MI and AFP, and the prognostic importance of Ki-67, MI and CGA in a cohort of patients treated, first line, with a single chemotherapy regimen. Accurate reporting of patient and tumour characteristics is therefore essential but randomised trials are also required to define the optimal regimen. In the UK, the most widely used combination chemotherapy for NETs is streptozocin and a fluoropyramidine (SF) and, on the basis of the data presented here, the UKCRN is currently recruiting to a randomised phase II trial of SF with/without cisplatin.

## Figures and Tables

**Table 1:** Clinicopathological features of patients

**Primary site**	**Pancreatic**	**Non-pancreatic**	**All**
Number	49	33	82
Median age (range)	54.9 (24.4–81.6)	57.4 (22.2–67.7)	55.4 (22.2–81.6)
Male	27	15	42
Female	22	18	40
			
*Performance status*			
0–1	44	27	71
⩾2	5	6	11
			
*Site*			
Lung	—	8	—
GI tract	—	9	—
Ovarian	—	1	—
Unknown	—	15	—
			
*Differentiation*			
Well	36	21	57
Poorly	9	9	18
			
*Grade*			
Low (1)	4	4	8
Intermediate (2)	31	16	47
High (3)	5	10	15
Unknown	9	3	12
			
Sites of metastases			
None	5	4	9
Liver	41	21	62
Lymph node	13	14	27
Lung	7	7	14
Bone	6	5	11
Other	10	6	16
			
*Prior systemic therapy*			
None	33	24	57
Radionucleotide	4	3	7
Embolisation	3	0	3
Interferon	2	1	3
Octreotide	8	7	15
Radiotherapy	0	1	1
			
*Octreotide scintigraphy*			
Positive	39	18	57
Negative	9	11	20
			
*5HIAA urine*			
Positive	4	7	11
Negative	21	16	37
			
*Chromogranin A*			
Positive (>60 U l^−1^)	17	10	27
Negative (≤ 60 U l^−1^)	9	10	19
			
*Tumour type*			
Non-functional	38	31	69
Gastrinoma	5	0	5
Glucagonoma	4	1	5
Somatostatinoma	1	1	2
Vipoma	1	0	1

Abbreviations: GI=gastrointestinal; 5HIAA=5-hydroxyindoleacetic acid.

**Table 2:** Toxicity according to CTCAE version 3.0

	**Worst toxicity grade (no of patients)**			
**Toxicity**	**1**	**2**	**3**	**4**
Nausea	23	22	10	2
Vomiting	12	15	12	2
Fatigue	25	29	6	0
Diarrhoea	12	9	6	2
Neuropathy	23	5	1	1
Stomatitis	17	7	1	0
Infection	6	8	3	3
ANC	8	19	21	2
Haemoglobin	27	16	6	0
Renal	5	5	0	0
Platelet	21	7	3	1

Abbreviation: ANC=absolute neutrophil count.

**Table 3:** Response and predictive markers

	**Response**					
**Predictor (normal range)**	**PR**	**SD**	**PD**	**PR (%)**	**Fisher's exact test *P*-value**	***χ*^2^-test for trend *P*-value**
All (*n*=79)	26	40	13	33		
						
*Primary*						
Pancreas	18	24	5	38	0.24^*^	—
Non-pancreas	8	16	8	25		—
Unknown	3	7	4	21		
Lung	2	4	2	25		
GI tract	2	5	2	22		
Ovarian	1	0	0	100		
						
*Octreotide*						
Positive	19	32	5	34	0.99	—
Negative	6	5	7	33		—
						
*5HIAA*						
Positive	2	6	2	20	0.99	—
Negative	10	19	7	28		—
						
*AFP (0–11.3 ng ml* ^*−1*^ *)*						
⩽16	10	22	8	25	0.020	—
>16	9	5	0	64		
						
*HCG (0–2.5 mIU ml* ^*−1*^ *)*						
⩽4	10	16	5	32	0.48	—
>4	5	5	1	46		—
						
*CA19.9 (0–39 U ml* ^*−1*^ *)*						
⩽40	7	17	5	24	0.23	—
>40	10	10	3	43		
						
*Chromogranin A (0–60 U l* ^*−1*^ *)*						
⩽60	6	12	1	32	0.99	
>60	9	13	3	36		
						
*Ki-67 (%)*						
⩽9	4	15	3	18	0.059	0.019
10–24	9	13	2	38		
⩾25	12	6	5	52		
						
*Mitosis per 10 HPF*						
0–1	3	14	3	15	0.032	0.008
2–4	7	13	4	29		
⩾5	11	5	4	55		
						
*WHO grade*						
1	1	4	2	14	0.085	0.023
2	15	25	6	33		
3	9	3	3	60		
						
*PS*						
0–1	22	36	10	32	0.99	
⩾2	4	4	3	36		
						
*Age*						
⩽60	20	27	10	35	0.60	
>60	6	13	3	27		
						
*Differentiation*						
Well	15	30	9	26	0.046	
Poorly	10	4	4	56		

Abbreviations: GI=gastrointestinal; 5HIAA=5-hydroxyindoleacetic acid; HCG=human chorionic gonadotropin; HPF=high-power field; PD=progressive disease; PR=partial response; PS=performance status; SD=stable disease; WHO=World Health Organization. ^*^Comparing pancreatic *vs* non-pancreatic.

**Table 4:** Univariable analysis for prognostic indicators

**Risk factor**	**PFS HR (99% CI)**	***P*-value**	**OS HR (99% CI)**	***P*-value**
Age (for an increase of 10 years)	1.05 (0.80– 1.39)	0.63	1.15 (0.84–1.59)	0.24
				
*Grade*				
1	1.00	0.39	1.00	0.03
2	0.73 (0.27–1.99)		0.45 (0.15–1.36)	
3	1.09 (0.34–3.44)		1.03 (0.30–3.52)	
				
*PS*				
0–1	1.00	0.33	1.00	0.038
>1	1.40 (0.59–3.28)		2.32 (0.89–6.05)	
				
*Primary site*				
Non-pancreatic	1.00	0.18	1.00	0.03
Pancreatic	0.72 (0.38–1.35)		0.54 (0.26–1.11)	
				
*Differentiation*				
Well	1.00	0.44	1.00	0.016
Poorly	1.26 (0.60–2.67)		2.16 (0.99–4.71)	

Abbreviations: HR=hazard ratio; OS=overall survival; PFS=progression-free survival; PS=performance status.

**Table 5:** Multivariable analysis for prognostic markers

**Marker**	**PFS HR (99% CI)**	***P*-value**	**OS HR (99%CI)**	***P*-value**
*AFP (*n*=57)*				
⩽16 ng ml^−1^	1.00	0.49	1.00	0.58
>16 ng ml^−1^	1.30 (0.50–3.42)		1.25 (0.45–3.52)	
				
*HCG (*n*=45)*				
⩽4 mIU ml^−1^	1.00	0.17	1.00	0.15
>4 mIU ml^−1^	0.72 (0.21–2.45)		2.35 (0.54–10.21)	
				
*CA19.9 (*n*=55)*				
⩽40	1.00	0.06	1.00	0.60
>40	0.49 (0.18–1.35)		0.81 (0.28–2.33)	
				
*Chromogranin A (*n*=46)*				
⩽60 U l^−1^	1.00	0.017	1.00	<0.001
>60 U l^−1^	2.77 (0.88–8.66)		6.77 (1.26–36.50)	
				
*Ki-67 % (*n=72)*				
<9	1.00	0.38	1.00	0.017
10–24	1.06 (0.43–2.59)		1.18 (0.39–3.55)	
⩾25	1.51 (0.65–3.51)		2.59 (0.98–6.83)	
				
*Mitosis per 10 HPF (*n=66)*				
0–1	1.00	0.04	1.00	0.017
2–4	1.04 (0.43–2.51)		1.29 (0.49–3.36)	
⩾5	2.29 (0.91–5.78)		2.99 (1.07–8.36)	

Abbreviations: AFP=*α*-fetoprotein; HCG=human chorionic gonadotropin; HPF=high-power field; HR=hazard ratio; OS=overall survival; PFS=progression-free survival.

Hazard ratios adjusted for age, grade, primary site and performance status. ^*^Adjusted for performance status, primary site and age.
